# Prevention of post-splenectomy sepsis in patients with asplenia - a study protocol of a controlled trial

**DOI:** 10.1186/s12879-019-4752-2

**Published:** 2020-01-14

**Authors:** Marianne Bayrhuber, Natascha Anka, Johannes Camp, Manuela Glattacker, Erik Farin, Siegbert Rieg

**Affiliations:** 1 Section of Health Care Research and Rehabilitation Research, Faculty of Medicine, University of Freiburg, Freiburg, Germany; 2 Division of Infectious Diseases, Department of Medicine II, Medical Center –Faculty of Medicine, University of Freiburg, Freiburg, Germany

**Keywords:** Asplenia, Post-splenectomy sepsis, Overwhelming post-splenectomy infection, Telephone intervention, Sepsis, Splenectomy, HAPA, Vaccination, Prevention

## Abstract

**Background:**

Patients with asplenia have a significantly increased lifelong risk of severe invasive infections, particular post-splenectomy sepsis (PSS). Clear preventive measures have been described in the literature, but previous studies found poor implementation of prevention recommendations. Aim of the study is to improve the adherence to guideline-based preventive measures and thereby reduce the incidence of PSS by a novel telephone-delivered intervention that involves both patients and their physicians.

**Methods:**

A prospective controlled, two-armed historical control group design is used to evaluate the new intervention compared to usual care. The intervention for patients includes both educational aspects and, building on the Health Action Process Approach (HAPA), intervention components that promote motivation and planning of preventive measures. For physicians the intervention is primarily information-based. The primary outcome, the adherence to preventative measures, is indicated by a study-specific ‘Preventing PSS-score’ (PrePSS-score), which is assessed at baseline and at 6-months follow-up. Secondary outcomes include, amongst others, patient self-efficacy and action-planning, asplenia-specific health literacy, general self-management and asplenia-specific self-management. In a process-evaluating part of the study interview-data on patients’ and physicians’ evaluation of the intervention will be gathered.

**Discussion:**

This trial will provide evidence about the effectiveness of the novel prevention intervention for asplenic patients. If demonstrated beneficial, the intervention manual will be made publicly available to enable implementation in practice. The experience gained within this trial may also be valuable for prevention strategies in patients with other diseases.

**Trial registration:**

German Clinical Trials Register (DRKS): DRKS00015238; Trial registration date 07. December 2018.

## Background

The spleen is the largest lymphatic organ and plays a crucial role in linking innate and adaptive immunity. As a result, the absence of the spleen is associated with significant morbidity and mortality [1]. Patients with anatomical asplenia (partial or total surgical removal of the spleen) or functional asplenia (loss of function of the spleen) have a significantly increased lifelong risk of severe invasive infections [2, 3]. The mortality of post-splenectomy sepsis (PSS, also called overwhelming post-splenectomy infection [OPSI]), the most dangerous complication, reaches 30–50% [4]. Studies report incidence rates of 7–8 infections requiring hospitalization per 100 patient-years and a post-splenectomy sepsis incidence of 1 per 100 patient-years. Compared to the general population, patients with asplenia have an approximately 6-fold increased risk of sepsis-related hospitalization [5].

The high mortality of these infections has led to guidelines for the prevention of sepsis in asplenic and hyposplenic patients. These recommendations include patient education, vaccinations, prophylactic and stand-by antibiotics, medical alert cards, travel advice and early treatment of animal bites [6, 7]. Patients without a functioning spleen and their physicians should be educated about the everyday risk of overwhelming infections and the need of prompt recognition and treatment of infections.

Asplenic patients should receive sequential pneumococcal vaccination (13-valent conjugate followed by 23-valent polysaccharide vaccine), meningococcal vaccination (tetravalent ACWY and serotype B vaccine), *Haemophilus influenza* type b conjugate vaccine and yearly influenza vaccination. A stand-by antibiotic should be prescribed for emergency use (‘pill in the pocket’). A smaller subgroup of patients (age < 5 years, patients after a PSS episode) should obtain antibiotic prophylaxis, although there is no international consensus on when to discontinue prophylaxis. Patients should carry a medical alert card that can inform physicians of the patient’s asplenia, optimally. Furthermore, travellers to high-risk areas, for example with regard to malaria, should secure optimal preventive measures. The effectiveness of these prevention measures has been shown in several studies [8–10].

Nevertheless, despite from these clear recommendations, previous studies have found poor adherence to preventive measures [2, 11]. In a recent prospective multicenter cohort study from Germany [12], the vaccination status was queried in patients with PSS admitted to an intensive care unit. Only 21% of patients had been vaccinated in the past 5 years according to the recommendations for asplenic patients with a pneumococcal vaccine; only 6% had ever been vaccinated against meningococci and 12% against *H. influenzae*. Accordingly, only 12% of patients had received a seasonal influenza vaccination. In the asplenia registry study at the Medical Centre University of Freiburg, only 6% of patients treated as part of regular care by general practitioners had completed the vaccination schedule according to current recommendations [12]. The registry data also show impressively that the booster immunization rates for each vaccine are again significantly worse than the primary immunization rates. In addition, only a minority (47%) of patients had received prescriptions for stand-by antibiotics.

Reasons for the lack of adherence to recommended prevention measures could be, on the one hand, that the prevention measures are unknown to patients and physicians [8]. On the other hand, patients might not be aware of the increased risk of infections, which could explain the low adherence to preventive measures. Several studies suggest the ‘Health Action Process Approach’ (HAPA) as a theoretical framework for the understanding of health behaviour in general [13–15] and for vaccination behaviour in particular [16–18]. The HAPA postulates a two-phase approach to action: Firstly, a pre-intentional motivational phase, which is characterized by risk perception, expectation of action results and expectation of self-efficacy and leads to an intention. And secondly, a post-intentional volition phase, which comprises factors as planning, action control, social support, recovery self-efficacy and leads to the actual health behaviour. Situational barriers and resources also play a role here as they influence the intention, planning and health behaviour. Social support, for example, represents a resource and the lack of it could be a barrier to adopt and maintain health behaviour [19]. Interventions to improve health behaviour beyond the passive provision of information material have not yet been described for asplenic patients.

Aim of the study is to improve the adherence to guideline-based preventive measures and thereby reduce the incidence of PSS by a novel telephone-based intervention that involves both patients and their general practitioners. By educating patients, the intervention contributes to the participation and empowerment of patients, who take responsibility for their own health in general and the implementation of prevention measures in particular. The new intervention is supposed to improve patients’ health by reducing morbidity as well as mortality and increase the quality-of-life of patients with asplenia. In addition, it can be expected that the costs of health insurance companies will decrease, since the treatment and follow-up costs of post-splenectomy infections are relatively high compared to the planned intervention and implementation of preventive measures. Evidence for this assumption can be found in cost-effectiveness analyses of PSS prevention in asplenia registries [12, 20]. Furthermore, the development of such an intervention can serve as a model for other studies.

Our assumption is that a targeted intervention strategy increases the adherence to recommended prevention measures.

## Methods/ design

### Aims and hypotheses

The purpose of this study is to develop, manualize and evaluate a novel intervention that educates both patients and their physicians on appropriate preventive measures that should be undertaken to prevent infections after splenectomy. Besides information provision, the intervention is intended to motivate patients to implement the preventive measures and to convey action-related skills such as planning and managing barriers. It will be evaluated whether this targeted intervention (intervention group) is superior to usual care (historical control group) in terms of primary and secondary study outcomes. More precisely, we have put forward the following hypotheses as to the outcome of the intervention: (a) Adherence to infection-risk reducing preventive measures will significantly be increased (primary outcome). As a result, (b) the incidence of severe infections associated with asplenia (particular PSS) and (c) related health-care costs covered by health insurances will be reduced (distal secondary outcomes). (d) Risk perception, intention to implementation, perceived self-efficacy, action and coping-planning, positive and negative outcome expectations and received social support (HAPA-related variables) expected to account for the effect of the intervention will significantly be enhanced (proximal secondary outcomes). Furthermore, (e) disease knowledge, patients’ general and asplenia-specific self-management, asplenia-specific health literacy, patient involvement as well as health-related quality of life will significantly be enhanced (distal secondary outcomes).

Beside this quantitative outcome-evaluation, intervention patients’ and intervention patients’ physicians’ acceptance and evaluation of the intervention will be inquired in telephone interviews in a process-evaluating part of the study.

### Study design and setting

**Fig. 1:**
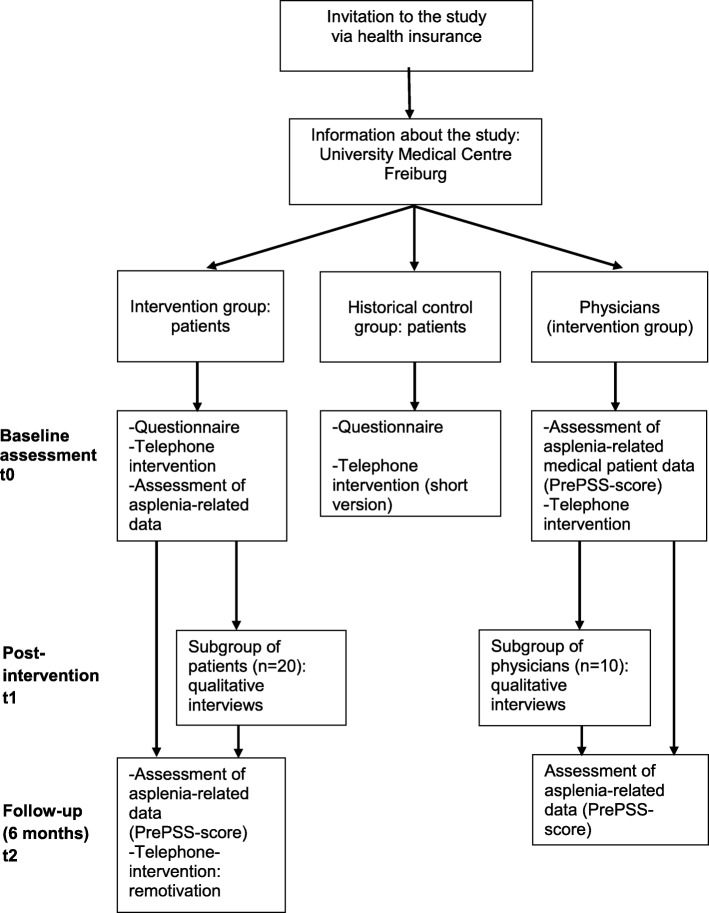
Flow chart of the study-design

This intervention study is designed as a prospective controlled, two-armed historical control group trial with baseline, post- and follow-up measurement and process evaluation (Fig. 1). The combination of outcome and process evaluation meets the recommendations for evaluating complex interventions [21]. As delaying the delivery of information on preventive measures puts patients on a non-justifiable risk [8] we decided against a randomized design and opted for a design with a historical control group for ethical reasons. In addition, the historical control group optimally illustrates current practice (‘usual care’).

The study is conducted by the Medical Center - University of Freiburg, Germany (Division of Infectious Diseases, Department of Medicine II and Section of Health Care Research and Rehabilitation Research, SEVERA) and the AOK Baden-Wuerttemberg, Germany.

### Intervention

The intervention comprises a telephone-based individual intervention session for patients with asplenia and a separate intervention for their physician, conducted by study physicians of the Medical Center - University of Freiburg with expertise in clinical infectious diseases. The content of the intervention was developed based on comprehensive literature review, existing guidelines for infection prevention and the study physicians’ expert knowledge. Both, the intervention sessions for patients and for their physicians are manual-guided to ensure a consistent practice across all study physicians; however, the interview protocol is semi-structured to allow an individualized proceeding. When developing the manual, particular attention was paid to its practical feasibility to enable a future implication beyond this study.

Prior to the implementation of the intervention, all participants are sent patient or physician tailored educational materials with brief information on the prophylaxis options along with a comprehensibly prepared vaccination plan and a medical alert card for patients with asplenia (see Additional file 1). It was developed by the Medical Center – University of Freiburg, the German Society of Infectious Diseases and the German Sepsis Society.

#### Patient-directed intervention (intervention group)

The 20-min intervention session for patients in the intervention group is divided into an information-giving section and, following the HAPA theory, intervention components that promote motivation for initiation (risk perception, positive outcome expectancies and task self-efficacy) and planning (action and coping planning, maintenance self-efficacy) of recommended infection prevention measures. Applied behavioral change techniques according to Abraham and Michie [22] comprise: providing information about behavior-health link and on the benefits of preventive measures, providing instruction, prompting intention formation, specific goal setting and barrier identification, assisting with relapse prevention by teaching to use prompts or cues or plan social support and use of follow-up prompts.

In the first section patients are provided with evidence-based information on the immunological function of the spleen, on potential infections after the spleen has been removed (targeting risk perception) and are educated on the most important preventive measures (targeting positive outcome expectancies). These comprise receiving asplenia-specific vaccinations (pneumococcal, meningococcal and *Haemophilus influenza* type b) and annual influenza vaccinations. Moreover, patients are advised to have an emergency supply of ‘pill in the pocket’-antibiotics to be taken in the event of sudden illness. The medical alert card for asplenic patients that informs health professionals about the splenectomy is introduced to them.

In the motivational-section patients are informed about the efficacy of the recommended preventive measures by means of a brief example on morbidity rates found to be higher among asplenic patients who are presumably unaware of their increased infection risk than among patients who received preventive education (targeting risk perception and positive outcome expectancies). Participants are sensitized to signs and symptoms that may indicate infection and are educated about the need of seeking rapid medical attention or taking emergency stand-by antibiotics if residing far from medical care and symptoms of infection occur (targeting task self-efficacy). In doing so, the information is framed in a way as to increase the awareness of the patient’s personal relevance rather than arousing fear of disease and it is focused on the feasibility of the recommended prevention behavior [23]. After explanation, patients are encouraged to determine which recommendations they want to follow by ticking corresponding boxes on their worksheets to prompt goal-setting.

In the planning-section patients are told that the aim is to facilitate implementation of the previously set prevention goals by precise planning. They are asked to develop action plans defining when, where and how they would take the intended infection preventive measures, including necessary preparatory behaviors, e.g. making appointments, fill in the medical alert card by physician (targeting action planning). Beyond that, participants are prompted to anticipate potential personal barriers to implementation of their personal plans. Amongst others, they are encouraged to think of situations in which the medical alert card could presumably be forgotten or circumstances that may led them failing to complete a vaccination course. At the same time, patients are assisted to find ways to attain their goals despite the identified impediments, for instance by seeking support from their networks (targeting maintenance self-efficacy and coping planning). To promote transfer into participants’ everyday lives they are encouraged to record their individual action and coping plans on designated worksheets accompanying the session.

At the follow-up telephone call, all planned preventive measures are assessed in order to calculate the PrePSS-score and, when indicated, potential barriers to implement plans are discussed. If further assistance for implementation is assumed, participants are re-motivated and assisted to manage difficulties. Follow-up consultation is optional and individually tailored to the patients’ needs and not manual-based.

#### Short version of the patient-directed intervention (historical control group)

Patients in the historical control group receive a shortened version of the patient-directed intervention. The short version includes the information-giving section on the functioning of the spleen and on health implications of asplenia (targeting risk perception, outcome expectancy and task self-efficacy) as well as the motivational-section on the efficacy of the recommended precautions and on strategies for risk situations (targeting positive outcome expectancy and task self-efficacy) since it is important for ethical reasons that the control group is provided with the same precautionary information as the intervention group. However, the planning-section is absent from the control group’s intervention as specific action plans and potential barriers are not discussed due to a lack of time resources. The intervention in the control group is implemented only for ethical reasons and is not an intervention variant to be evaluated. Data collection relevant for the study has already been completed in this group at the time of intervention implementation, thus a confounding influence on the outcome variables can be ruled out.

#### Physician-directed intervention

The physician-directed telephone intervention comprises evidence-based information on the consequences of asplenia and the increased infection risk associated with high mortality rates. Physicians are educated about preventive measures consistent with current post-splenectomy guidelines including currently recommended vaccination and revaccination on the basis of given immunization schedules, indication of stand-by antibiotics and antibiotic prophylaxis. Furthermore, the intervention session includes an introduction in the purpose and use of the medical alert card and the necessity of patient education, particularly as to the patient-initiated antibiotic use in case of febrile illness. Physicians are advised to document any antibiotics in use and record a vaccine plan (vaccination status, need and interval for revaccination) specific to their patient using provided fields on the medical alert card.

The objective of the intervention is to heighten physicians’ awareness and knowledge of available preventive measures to improve guideline-based post-splenectomy care. Thereby the information-provision component is the integral part of the physician’s intervention. However, in line with the patients’ intervention, the physicians’ intervention also targets risk perception, positive outcome expectancy and, subsequently, motivation to follow guideline recommendations.

The intervention sessions take approximately 10 min. All participating physicians receive the intervention irrespective of the group allocation of their patients.

### Participants and recruitment

Participants are patients with anatomic asplenia and their physicians (general practitioners or specialists). Eligible are German-speaking patients aged 18 years or older, who are insured by the cooperating health insurance AOK Baden-Wuerttemberg, which is Germany’s 5th largest health insurance and insures more than 4 million people.

Patient participants are preselected to either the intervention or control group based on the time interval since they underwent splenectomy. Patients who are recently splenectomized (at most 4 months) are allocated to the intervention group. Potential intervention patients are recruited about 6–8 weeks after splenectomy successively by biweekly request from February 2019 for a maximum period of 18 months. The historical control group consists of patients who are splenectomized since more than 6 months (at most 18 months). Thus, pre-interventional baseline-data on primary and secondary outcomes in the historical control group account for routine care. Potential control group patients were recruited at the start date of study implementation (January 2019) and, to attain the planned sample size, another cohort of patients was contacted half a year later (June 2019).

Potential patient participants are identified via a database search (search criteria: OPS-code 5–413 splenectomy, with all sub-codes 5–413.0 [partial splenectomy] and 5–413.1 [total splenectomy]) for all splenectomized patients within the predefined group-specific time periods since splenectomy by the AOK-Baden-Wuerttemberg. Patients who meet criteria receive recruitment letters from the health insurance. Those who are interested in study participation receive detailed information on the procedure, the aims and the legal conditions of the study from the University Medical Centre Freiburg. Participants are asked to provide written informed consent and contact information if they agree to take part.

To identify the corresponding physician, participating patients are asked to provide contact information on their general practitioner or other physician who mainly cares for their asplenia and sign an agreement releasing the physician form medical confidentially obligation. Physicians whose patients consent to having their physicians included in the study are recruited by letters with information concerning the study. No exclusion criteria for physicians are applied. Both patient and physicians will receive a 30€ voucher for participation after study completion.

### Sample size considerations

A priori calculation of the sample size of patient participants to compare the intervention group to the historical control group in the primary outcome was performed with the software ‘*Power and Precision*’*.* Based on an assumed medium to large effect size of 0.40, a statistical power of 80% and a significance level of 5% (two-sided) the minimum required patient sample of *N* = 100 per group was calculated. Further sample size considerations take into account the actual number of splenectomized patients insured by the AOK. An explorative request showed that 360–400 patients undergo splenectomy a year, resulting in approximately 500 patients assumed to be available for recruitment in the planned inclusion period of 18 months. Based on studies with similar patients [24], we further estimated a proportion of 50% non-respondents for the intervention group and attrition rates of 40% of respondents, so we aim to recruit 500 potential intervention group patients for an expected analysis sample of *N* = 178 participants. As this exceeds the statistically required number of cases despite conservative estimations, sufficient cases will be available even after considering potential deceases in the course of the study.

Given that the inclusion period (18 months) is the same for the control group (although retrospectively), sample size considerations for this group are largely the same, with the exception of an assumed proportion of 60% non-respondents and a dropout rate of 50%, resulting in expected *N* = 110 control group cases for analysis. Correspondingly, the number of physician participants included in analysis will be *N* = 178 intervention patients’ physicians and *N* = 110 control patients’ physicians maximum, considering that some patients may have the same physician.

### Outcome measures

#### Primary outcome

*The primary outcome*, the adherence to preventative measures, is indicated by a study-specific ‘Preventing PSS-score’ (PrePSS-score), which includes the following parameters: (a) receipt of guideline-conform sequential pneumococcal vaccination and (b) guideline-conform meningococcal vaccinations, (c) prescription and availability of stand-by antibiotics for emergency treatment and (d) handing out of and carrying a medical alert card for asplenic patients. The selection of the included parameters was made by Infectious Diseases specialists of the University Medical Center Freiburg based on current guidelines and recommendations on PSS prevention [25, 26].

To weight these preselected parameters, an expert survey was conducted prior to the study asking a total of 16 international experts in the care of asplenic patients to rate the items according to their importance in infection prevention, of which nine experts provided feedback. Based on the calculated median of the given expert-ratings the scoring system was defined (see Table 1 for an overview, for exact score formation and operationalization see Additional files 2 and 3).

The score for each patient is estimated by the study physicians according to both the patient’s and the physician’s information gathered through telephone-interviewing. To validate the self-report data on the primary outcome, health insurance patient routine data aggregated by groups (vaccinations relevant to asplenia and prescribed antibiotics) will be included.

**Table 1:** Parameters and scoring system of the PrePSS-score

Parameter	Score
(1) Guideline-conform sequential pneumococcal vaccination	0–3
(2) Guideline-conform meningococcal vaccination	0–3
(3) Stand by-antibiotic prescribed and available (‘pill in the pocket’)	0–2
(4) Handing-over and carrying a medical alert card	0–2
Total PrePSS-score [Range]	0–10

#### Secondary outcomes

In this article secondary patient outcomes are classified on a proximal-distal continuum of outcome measures [27]. HAPA-related variables (i.e. patient’s risk perception, self-efficacy and action-planning) are considered as *proximal secondary outcomes* which are assumed to be more likely and directly affected by the intervention than distal outcomes and observable shortly after the intervention. More global, *distal secondary outcomes,* are assumed to be also influenced by proximal outcomes as well as external, non-treatment factors. These include disease knowledge (disease knowledge is also expected to be a confounder), patient general and asplenia-specific self-management as well as asplenia-specific health literacy, self-reported patient involvement and health-related quality of life.

Secondary outcomes for physician participants are their subjective improvement in knowledge and their satisfaction with the intervention.

### Questionnaires

Patient-related secondary outcome measures, potentially confounding variables and the patients’ evaluation of the telephone-intervention are assessed via self-administered paper-pencil questionnaires incorporating already validated instruments as well as asplenia-specific scales developed for the purpose of this study, which are described below.

1. **HAPA-related outcomes.** To gather key HAPA variables addressed in the intervention, perceived disease risk relevant to asplenia, patients’ behavioral intention to implementation, perceived self-efficacy for implementation, action and coping planning, positive and negative outcome expectations and received social support are assessed. Items were developed on the basis of the general assessment rules for HAPA constructs provided by Schwarzer et al. [19] and slightly adapted to infection prevention behavior in asplenia. Responses are rated on a six-point scale ranging from *fully correct* (1) to *not correct at all* (6) (except the scale *risk perception*).
  Six items measuring *behavioral intention* to obtain prevention refer, for instance, to “undertake preventive measures recommended after splenectomy” and “obtain vaccinations”. *Perceived self-efficacy* is assessed by ten items asking participants to rate their level of confidence in their ability to implement and cope with preventive measures, such as “I can correctly interpret symptoms of a severe infection” or “I will renew my emergency antibiotics after the expiration date”. *Prevention behavior planning* is assessed with six items, four items measuring action planning as the items address the where and how of the precautions (e.g. “what kind of vaccinations I will get done”) and two items measuring coping planning asking for situations that could interfere with their plans (e.g. “what I can do if I forget my emergency antibiotics”). *Positive and negative outcome expectancies* after implementing the preventive measures are assessed with three items asking for pros, e.g. “I’m better protected from the flu” and three items asking for cons, e.g. “I could suffer from side effects of vaccinations”. *Received social support* regarding prevention implementation is measured with the stem “People around me (e.g. family, friends)…” followed by five items, for example “have encouraged me to take preventive measures recommended after splenectomy”. For *risk perception*, the item stem “If I compare myself with other people (of my age and sex), then my risk, sometime in future…” is followed by the items “to fall ill with blood poisoning”, “to fall ill with meningitis” and “to get pneumonia”, which are rated by participants on a scale from *significantly increased* (1) to *considerably lower* (5).
2. **Self-management.** General self-management is assessed with the two subscales *Self-Monitoring and Insight* and *Skill and Technique Acquisition* from the German version of the Health Education Impact Questionnaire (heiQ) [28, 29], a widespread tool developed to assess proximal outcomes of patient self-management programs, covering eight independent dimensions. The scale *Self-Monitoring and Insight* (six items) captures individuals’ ability to monitor their condition that leads to insight and appropriate actions to self-manage as well as individuals’ acknowledgment of realistic disease-related limitations. The scale *Skill and Technique Acquisition* (four items) covers the subjective appraisal of knowledge-based skills and techniques that help manage disease-related symptoms and health problems. Items are scored on a 4-point response scale (1 = *strongly disagree* to 4 = *strongly agree*) and averaged for the two scales, with higher values indicating a higher subjective judgement of self-monitoring and skills respectively.
3. **Asplenia-specific self-management and asplenia-specific health literacy.** To capture disease-specific self-management components, five items related to asplenia were developed on the basis of the heiQ-scales *Self-Monitoring and Insight* (three items) as well as *Skill and Technique Acquisition* (two items), described above. One item each derived from the heiQ-scales *Health-Service Navigation* and *Social Integration and Support* were used to develop two further items as these aspects are additionally relevant for asplenia-specific self-management.
  A total of six items capturing asplenia-specific health literacy were derived from the Health Literacy Questionnaire (HLQ) [30]. The HLQ covers nine health literacy domains that reflect an individual’s competencies and experiences when attempting to understand, access and use health-information or when trying to engage with healthcare practitioners or services, of which five were used as basis for the development of the disease-specific health-literacy items. The response format for all self-developed items is a 6-point scale ranging from *fully correct* (1) to *not correct at all* (6).
4. **Patient involvement*.*** Patient involvement is measured with the German version of the Perceived Involvement in Care Scales (PICS) [31, 32] a 14-item generic instrument that is designed to assess patients’ perceptions of participation in treatment decision making as well as physicians’ efforts to facilitate patient involvement. It covers three categories of patient-physician communication: *Doctor Facilitation of Patient Involvement, Level of Information Exchange* and *Patient Participation in Decision Making*. The response scale is a 4-point Likert scale ranging from 1 (*strongly disagree*) to 4 (*strongly agree*), where higher scores indicate higher perceived patient activity and endorsement.
5. **Health-related quality of life.** The 12-item Short-Form-Health-Survey (SF-12, short version of SF-36) is administered to assess self-reported health-related quality of life referring to the past 4 weeks [33]. The SF-12 is a generic instrument that yields a subjective mental and physical health status summary score derived from four health components respectively: Physical health comprises *general health, physical functioning, role limitations due to physical health problems* and *bodily pain*; subjective mental health comprises *vitality (energy/fatigue), social functioning, role limitations due to emotional problems* and *mental health*. High scale values indicate better health.
6. **Subjective and objective disease knowledge**. Subjective disease knowledge held by patients is assessed using two items asking them to rate their level of knowledge about the consequences of splenectomy and potential preventive measures on a 5-point scale (1 = *very great knowledge* to 5 = *non-existent knowledge*). Four additional questions are administered to ascertain the objective degree of knowledge about asplenism. The items refer to the functions of the spleen, consequences of splenectomy, recommended precautions and patients theoretically behavior in case of sudden septic symptoms (of which the latter is derived from Gundling et al. [33]).
7. **Compliance and influenza prevention behaviors.** Patients’ compliance with general health-preserving measures is estimated using four items of the German version of the Questionnaire of Multiple Health Behavior (MHB-39) [34] that load highest onto the domain *Compliance* (i.e. having regular check-ups and prophylactic vaccinations made, complying with physicians and consulting a doctor when indicated). The MHB-39 assesses habitual health-related behaviors on a 5-point Likert scale (1 = *never* and 5 = *always*). In our questionnaire, the MHB-39 compliance-items are supplemented by three questions asking for patients influenza prevention behaviors (i.e., washing hands after return to home and before touching food, avoid touching eyes or mouth in public, avoid hand shaking during flu season) taken from Zhang et al. [16] and translated into German.
8. **Depression and anxiety.** Indicators of depression and anxiety in patient participants are measured using the German version of the Patient Health Questionnaire for Depression and Anxiety (PHQ-4) [35], a validated four-item ultra-brief screening instrument that consists of a 2-item *depression scale* (Patient-Health-Questionnaire, PHQ-2) [35, 36] asking for DSM-IV diagnostic core criteria symptoms (i.e. loss of interest, depressed mood) and a 2-item *anxiety-scale* (Generalized Anxiety Disorder Scale, GAD-2) [36] representing core symptoms of a generalized anxiety disorder (feeling nervous and anxious, difficulty to stop or control worrying). The stem question for all items is: “Over the last two weeks, how often have you been bothered by any of the following problems?”. Answers a given on a 4-point Likert-type scale ranging from *not at all* (0) to *nearly every day* (3). Scale scores ≥3 indicate the presence of a depression or an anxiety disorder, respectively.
9. **Evaluation of the telephone-intervention.** Patients are asked to judge patient-centered criteria of the telephone-intervention using six items relating to the content (i.e. topic selection, comprehensibility, and usefulness), materials and interaction (atmosphere, opportunity to make own comments or pose questions). Items are rated on a school grading scale ranging from (1) *very good* to (6) *very poor*. Two further open questions inquire positive feedback and suggestions for improvement. Items were taken from Meng et al. [37] and slightly adapted to our intervention.

### Procedure

The chosen outcomes for patients are measured prior to the intervention (baseline measurement, t0), directly after the intervention (t1) and after a 6-month follow-up period (t2).

At t0, patients in the intervention group are sent paper-pencil pseudonymized questionnaires on baseline proximal and distal secondary outcomes and socio-demographic information. Upon receipt of the filled questionnaires, telephone appointments are arranged with patients for a study physician interview. During telephone calls, a vaccination history, use and availability of stand-by antibiotics and the medical alert card are taken to gather baseline data on the primary outcome, the PrePSS-score, along with some other medical information relating to the patients’ asplenia (e.g. indication for splenectomy, splenectomy date and previous episodes of infection or PSS requiring hospitalization).

After t0-data collection, the patient-directed telephone-intervention is implemented. The intervention of the corresponding physician is conducted at about the same time; however, the order is determined by the arrangement of the telephone appointments and not standardized. Secondary physician outcomes are gathered after the physician-directed intervention. Historical control group patients and their physicians go through the same procedure and patients receive t0-questionnaires identical to intervention group patients, but only intervention participants continue measurement after the telephone intervention.

Following each patient telephone call, intervention patients complete post-intervention questionnaires on the proximal secondary outcomes similar to baseline items and evaluate the telephone-intervention (t1).

To test for six-month sustainability of the effects of the intervention they receive follow-up questionnaires on distal secondary outcomes identical to baseline measurement (t2). After return of the follow-up questionnaires, intervention patients are contacted by study physicians via telephone again to inquire the same set of data on the primary outcome and (changes in) medical data, such as the incidence of infections and PSS, gathered at t0.

To ensure a valid data basis, patients’ self-report data on the primary outcome variable and on the medical information are confirmed with the corresponding physician both at t0 and (in the intervention group) at t2. In case physicians are interviewed prior to their patients, patients are made aware of any discrepancies between their information and information their physicians provided when required.

### Qualitative interviews for process evaluation

A total of 20 patients of the intervention group and 10 intervention patients’ physicians (first patients or physicians who agree to participate) are surveyed 5.5 months after telephone-intervention (shortly before t2-measurement) in semi-structured 20- to 30-min telephone-interviews by psychologists of the project team. Patients are interviewed on their acceptance and perception of the telephone-intervention and accompanying materials as well as on the feasibility (e.g. experience in implementation, helpful factors and barriers) of intervention contents. Physicians are asked for their subjective evaluation of the intervention (e.g. usefulness, improvement suggestions). Interviews will be audio-recorded with the permission from participants.

### Data analysis

Demographic characteristics of the study population and effect sizes in the intervention group will be reported descriptively. The main analysis tests the hypothesis that the PrePSS-score at follow-up is higher (better adherence to infection-risk reducing preventive measures) in the intervention group than in the control group. Due to the non-randomized design a propensity score adjustment is performed to reduce potential bias that may be caused by differences on covariates in the two groups [38]. We will apply general linear models with propensity score as a covariate. The same method will be used for the analysis of secondary outcomes. Assuming missing data in the questionnaires, multiple imputation will be considered for corresponding analyses. Additional analyses will be conducted with structural equation modeling technique to test a priori specified mediation models of intervention effects.

A cost-effectiveness analysis of the intervention will be conducted by analyzing the change-from-baseline scores of the primary and the secondary outcomes in relation to the costs of the intervention. To reveal the economic efficiency of the intervention, routine data will be used to determine standard treatment and follow-up costs associated with infections requiring hospitalization and with PSS in asplenic patients to contrast them to the intervention costs.

In the qualitative analyses, the audio files of the interviews will be transcribed by an external service provider and the transcripts will be analyzed using a qualitative content analysis.

## Discussion

Poor implementation of the prevention recommendations for patients without a functioning spleen has been demonstrated in several studies. Better adherence to preventive measures is urgently needed [2, 11]. However, conclusive and effective new strategies to improve care beyond the passive provision of information have not yet been described for asplenic patients.

Strengths of the current study are the development and evaluation of a theory-based dual intervention, i.e. focusing on patients and their physicians. By educating and training patients, the intervention contributes to the empowerment of the patients. Quantitative data will allow us to evaluate the effect of the intervention on prevention measures such as vaccinations, prophylactic and stand-by antibiotic use and patient-related outcomes*.* Qualitative interviews will enable us to understand e.g. barriers in preventive behaviour. Furthermore, the new intervention can be improved on the basis of feedback from asplenic patients and their physicians. Following this evaluative process, the intervention-manual will be made publicly available to enable future implementation in practice.

The study has some limitations, which are mainly based on our sampling strategy. First, our sample contains a self-selected group of patients from the cooperating health insurance (AOK Baden-Wuerttemberg). Secondly, it is not a randomized controlled trial, however, due to above outlined ethical reasons randomization is not justifiable. In order to reduce a potential bias that may be caused by differences in covariates in the intervention vs. historical control group, propensity score matching will be applied. Third, the primary outcome, the PrePSS-score was developed via expert-ratings, however, weighting of the four included items may still need further refinement and research.

All in all, we believe that the experience gained with this type of intervention will also be very valuable for prevention strategies in patients with other diseases. The intervention could be considered - after demonstrated effectiveness - in the context of other poorly implemented primary prevention measures or standard vaccinations, e.g. influenza and pneumococcal vaccination in patients over the age of 60 years.

## Supplementary information

**Additional file 1.** Medical Alert Card Asplenia.

**Additional file 2: Table S2.** Results of the expert survey.

**Additional file 3: Table S3.** Operationalization of the PrePSS-score parameters after weighting of the parameters.

## Data Availability

Not applicable.

## Abbreviations

GAD-2: Generalized Anxiety Disorder Scale
HAPA: Health Action Process Approach
heiQ: Health Education Impact Questionnaire
HLQ: Health Literacy Questionnaire
MHB-39: Questionnaire of Multiple Health Behavior
OPSI: Overwhelming post-splenectomy infection
PHQ-2: Patient-Health-Questionnaire
PHQ-4: Patient Health Questionnaire for Depression and Anxiety
PICS: Perceived Involvement in Care Scales
PrePSS-score: Preventing PSS-score
PSS: Post-splenectomy sepsis
SF-12: 12-item Short-Form-Health-Survey
