# Supplementary material for: Prevention of post-splenectomy sepsis in patients with asplenia - a study protocol of a controlled trial
Source: BMC Infect Dis. 2020 Jan 14;20:41. doi: 10.1186/s12879-019-4752-2 (PMC6961276; doi:10.1186/s12879-019-4752-2)
Supplement: Supplementary file 1 — Additional file 1. Medical Alert Card Asplenia. [file 12879_2019_4752_MOESM1_ESM.pdf]

Indikationsimpfungen bei Asplenie

Datum	Pneumokokken		Meningokokken			Haemo-philus	Grippe	Unterschrift und Stempel
	PCV-13	PPV-23	MCV-C	MCV-ACWY	Men-B			

Antibiotikaprophylaxe

Präparat	von	bis	Präparat	von	bis

Für Abkürzungen siehe Impfplan auf der Rückseite

Im Auftrag und mit Unterstützung von:

Deutsche Gesellschaft für Infektiologie

Deutsche Gesellschaft für Pädiatrische Infektiologie

Deutsche Sepsisgesellschaft

Deutsche Gesellschaft für Chirurgie

Gesellschaft für Pädiatrische Onkologie und Hämatologie

Deutsche Sepsis-Hilfe

Impressum

Abteilung Infektiologie, Klinik für Innere Medizin II
und IFB-Zentrum Chronische Immundefizienz (CCI)
Universitätsklinikum Freiburg
Hugstetter Straße 55
D-79106 Freiburg im Breisgau
aspleniepass@asplenie-net.org
www.asplenie-net.org

Gefördert vom Bundesministerium für Bildung und Forschung

Gefördert durch Gemeinsamer Bundesausschuss Innovationsausschuss

4/2018

Bestellformulare: www.asplenie-net.org

NOTFALLAUSWEIS

Medical Emergency Card Asplenia

Asplenie

(Fehlende Milz / Milzfunktion)

Es besteht ein erhöhtes Risiko für lebensbedrohliche Infektionen !

Inhaber

Name	
Vorname	
Geburtsdatum	
Adresse	

Asplenie / Splenektomie

seit / am	

Im Notfall zu benachrichtigen

Name	
Telefon	

Schwere Antibiotikaallergie

nein ❑ ja ❑

gegen	
Stempel des Hausarztes oder Kinderarztes:	

Impfplan bei Asplenie

Bitte Aktualisierungen unter www.asplenie-net.org beachten! (Stand: 4 / 2018)

Alter	Grundimmunisierung	1. Auffrischimpfung	Weitere Auffrischimpfung
**Pneumokokken**			
2 Mo - 24 Mo.	gemäß Impfkalender STIKO	PSV-23 (nach 6-12 Mo.)	PSV-23 (nach 5-6. J.) **
3-5 J.	PCV-13 *	PSV-23 (im Alter von 6 J.)	PSV-23 (nach 5-6. J.) **
≥6 J. u. Erwachsene	1 x PCV-13	PSV-23 (nach 2-6 Mo.)	PSV-23 (nach 5-6. J.) **
* falls noch keine komplette Grundimmunisierung	** Notwendigkeit von PCV-13 Wiederauffrischung derzeit noch unklar		
**Meningokokken**			
2-11 Mo.	2 x Men-C (Abstand 2 Mo.)	Men-ACWY + (nach 12 Mo.)	Men-ACWY (nach 6-12 Mo.) ++
≥ 1 J. u. Erwachsene	1 x Men-ACWY +	Men-ACWY (nach 2 Mo.)	Men-ACWY (alle 5 J.)
+ Zulassung beachten: Nimenrix® ab 2. Lebensjahr; Menveo® ab 3. Lebensjahr;	++ spätere Auffrischungen mit MCV-ACWY alle 5 J.		
2-5 Mo.	3 x Men-B ‡ (Abstand 1 Mo.)	Men-B ‡ (nach 12 Mo.)	Notwendigkeit derzeit unklar
6-23 Mo.	2 x Men-B ‡ (Abstand 2 Mo.)	Men-B ‡ (Abstand s. u.) ‡‡	
2-10 J.	2 x Men-B ‡ (Abstand 2 Mo.)		
≥ 10 J. u. Erwachsene	2 x Men-B ‡ (Abstand 2 Mo.)	Men-B (Abstand 4 Mo.) ‡‡	
‡ Zulassung beachten: Trumenba® erst ab 10 J. einsetzbar.			
‡‡ Alter 6-11 Mo.: Auffrischung im2. LJ (Abstand 2 Mo.), Alter 12-23 Mo.: Abstand 12 Mo.	‡‡ erforderlich nur für Trumenba®		
**Haemophilus influenzae Typ B**			
2 Mo.-5 J.	Grundimmunisierung gemäß Impfkalender STIKO		
> 5 Jahre	Einmalige Impfung mit HiB-Konjugatimpfstoff		
**Influenza (Grippe)**			
≥ 2 Jahre	Jährliche Grippeschutzimpfung (aktuelle von der WHO empfohlene Antigenkombination)		

PCV-13	13-valenter Pneumokokken-Konjugatimpfstoff	Prevenar-13®
PSV-23	23-valenter Pneumokokken-Polysaccharidimpfstoff	Pneumovax®
Men-C	Meningokokken-Konjugatimpfstoff Serogruppe C	Meningitec®, NeisVac-C®, Menjugate®
Men-ACWY	Meningokokken-Konjugatimpfstoff Serogruppe A, C, W, Y	Menveo®, Nimenrix®
Men-B	Meningokokken-Konjugatimpfstoff Serogruppe B	Bexsero®, Trumenba®
HiB	*Haemophilus influenzae* Typ B-Konjugatimpfstoff	ACT-HiB® (nur über Import)

Notfallantibiotikatherapie – Optionen

**bei Kindern**	
p.o.<br>i.v.	Amoxicillin / Clavulansäure 3 x 25 mg/kg/KG<br>Ceftriaxon 1 x 80 mg/kg/KG
**bei Erwachsenen**	
p.o.<br>i.v.	Amoxicillin / Clavulansäure 3 x 1000 mg<br>Ceftriaxon 1 x 2000 mg
**bei Allergie**	
Penicillinallergie<br>β-Laktamallergie	Cefpodoximproxetil<br>Clarithromycin
Empfehlungen zur Antibiotikaprophylaxe siehe www.asplenie-net.org	

Erhöhtes Infektionsrisiko

Sepsis mit bekapselten Bakterien: Pneumokokken (häufig), Meningokokken, *H. influenzae*

Sepsis nach Tierbissen: *Capnocytophaga canimorsus*

Parasitosen: *Babesia* (Zeckenstiche), Malaria

Bei Fieber oder Infektionszeichen ist eine schnelle Antibiotikatherapie angezeigt !
