# Supplementary material for: Prevention of post-splenectomy sepsis in patients with asplenia - a study protocol of a controlled trial
Source: BMC Infect Dis. 2020 Jan 14;20:41. doi: 10.1186/s12879-019-4752-2 (PMC6961276; doi:10.1186/s12879-019-4752-2)
Supplement: Supplementary file 2 — Additional file 2: Table S2. Results of the expert survey. [file 12879_2019_4752_MOESM2_ESM.docx]

**Additional file 2: Table S2**: Results of the expert survey

| **Parameter** | **Expert**  **1** | **Expert**  **2** | **Expert**  **3** | **Expert**  **4** | **Expert**  **5** | **Expert**  **6** | **Expert**  **7** | **Expert**  **8** | **Expert**  **9** | **Median** | **Mean** | **Final result** |
| --- | --- | --- | --- | --- | --- | --- | --- | --- | --- | --- | --- | --- |
| **(1) Pneumococcal vaccination** | 3 | 1 | 3 | 3 | 3 | 3 | 3 | 3 | 3 | 3 | 2,78 | 3 |
| **(2) Meningococcal vaccination** | 2 | 1 | 3 | 3 | 3 | 3 | 3 | 2 | 3 | 3 | 2,56 | 3 |
| **(3) Stand by-Antibiotics** | 2 | 1 | 2 | 3 | 2 | 3 | 3 | 2 | 3 | 2 | 1,89 | 2 |
| **(4) Medical alert card** | 1 | 3 | 1 | 3 | 1 | 3 | 2 | 1 | 2 | 2 | 1,89 | 2 |
| **Total PrePSS**  **Score** | 8 | 6 | 9 | 12 | 9 | 12 | 11 | 8 | 11 | 10 | 9,12 | 10 |

Each expert was asked to rate the four preselected PrePSS-score parameters according to their importance in infection prevention in a range of 0 to 3 points. Based on the calculated median of the given expert-ratings the weighting of the PrePSS score was defined (last column).
