# Supplementary material for: Prevention of post-splenectomy sepsis in patients with asplenia - a study protocol of a controlled trial
Source: BMC Infect Dis. 2020 Jan 14;20:41. doi: 10.1186/s12879-019-4752-2 (PMC6961276; doi:10.1186/s12879-019-4752-2)
Supplement: Supplementary file 3 — Additional file 3: Table S3. Operationalization of the PrePSS-score parameters after weighting of the parameters. [file 12879_2019_4752_MOESM3_ESM.docx]

**Additional file 3: Table S3**: Operationalization of the PrePSS-score parameters after weighting of the parameters

| **Parameter** | **Score** |
| --- | --- |
| **(1) Guideline-conform sequential pneumococcal vaccination** | **Range 0-3** |
| - PCV-13 and PSV-23, correct sequence and intervals^1^ - PCV-13 and PSV-23, altered sequence or intervals - Only PCV-13 or PSV-23 - No pneumococcal vaccination | 3  2  1  0 |
| **(2) Guideline-conform meningococcal vaccination** | **Range 0-3** |
| - Men-ACWY and Men-B complete primary immunization^2^ - Men-ACWY and Men-B incomplete primary immunization - Men-ACWY or Men-B - No meningococcal vaccination | 3  2  1  0 |
| **(3) Stand by-antibiotic prescribed and available (‘pill in the pocket‘)** | **Range 0-2** |
| - Stand by-antibiotic prescribed, antibiotic permanently available (24 h/7 d) - Stand by-antibiotic prescribed, antibiotic not permanently available - No stand by-antibiotic prescribed | 2  1  0 |
| **(4) Handing-over and carrying a medical alert card** | **Range 0-2** |
| - Medical alert card handed out, permanently carried along (e.g. in purse) - Medical alert card handed out but not permanently carried along - No medical alert card handed out | 2  1  0 |
| **Total PrePSS-score [Range]** | **0-10** |

^1^ 13-valent conjugate vaccine PCV-13 (Prevenar-13^®^) after ≥2 months followed by 23-valent polysaccharide vaccine PSV-23 (Pneumovax^®^)

^2^ Tetravalent meningococcal conjugate vaccination Men-ACWY (Menveo^®^, Nimenrix^®^), two doses at least two months apart; meningococcal serotype B vaccine Men-B (Bexsero^®^ [two doses] or Trumenba^®^ [three doses]).
